# Study on maintenance of eyeball morphology by foldable capsular vitreous body in severe ocular trauma

**DOI:** 10.1186/s12886-023-03209-4

**Published:** 2023-11-16

**Authors:** Shanyu Li, Xiaoxuan Wang, Zhixia Dou, Jie Zhang, Jinchen Jia

**Affiliations:** 1 Hebei Provincial Key Laboratory of Ophthalmology, Hebei Provincial Clinical Research Center for Eye Diseases, Hebei Eye Hospital, 054000 Xingtai, Hebei China; 2 Xingtai Third Hospital, 054000 Xingtai, Hebei China

**Keywords:** Foldable capsular vitreous body, Severe case, Ocular trauma, Visual acuity, Intraocular pressure, Retina

## Abstract

**Objectives:**

To explore the feasibility and safety of using a foldable capsular vitreous body (FCVB) in managing severe ocular trauma and silicone oil-dependent eyes.

**Methodology:**

This is a retrospective study of 61 ocular trauma patients (61 eyes) who presented to the Department of Eye Emergency, Hebei Eye Hospital from May 1, 2018, to May 31, 2019, including 51 male patients (51 eyes) and 10 female patients (10 eyes) with an average age of 44.98 ± 14.60 years old. The oldest patient was 75 years old, and the youngest was 8 years old. These cases represented 51 eyes with severe eyeball rupture and 10 eyes with severe, complicated ocular trauma, which became silicone oil-dependent after the operation. These patients received FCVB implants, and data regarding their visual acuity, intraocular pressure, changes in eye axis, cornea, retina, and FCVB state were recorded after the operation.

**Results:**

In all patients, the FCVB was properly positioned and well supported with the retina. All 61 patients cleared a follow-up window of 1–36 months with no reports of important changes in their visual acuity. Among the patients, 91.8% reported normal intraocular pressure, the retinal reattachment rate reached 100%, and the eyeball atrophy control rate reached 100%. There was no report of rupture of the FCVB, allergies to silicone, intraocular infection, intraocular hemorrhage, silicone oil emulsification, or sympathetic ophthalmia.

**Conclusions:**

Foldable capsular vitreous bodies (FCVBs) designed to mimic natural vitreous bodies are suitable as long-term ocular implants that can provide sustained support for the retina without the need for any special postoperative postures. Their barrier function may effectively prolong the retention time of the tamponade and prevent various complications caused by direct contact of the eye tissues with the tamponade.

## Introduction

Ocular trauma is one of the main causes of visual impairment [1]. Severe cases of ocular trauma are generally associated with poor prognoses [2]. The most common causes of severe ocular trauma include injuries by impact, sharp objects, and explosions. In most cases, the posterior segments of the eyeball are involved. Severe trauma manifests in conditions including ruptured globes, intraocular foreign bodies, ocular penetration injuries, post-traumatic endophthalmitis, etc. [1, 2]. Severe ocular trauma poses an intractable problem to ophthalmologists, especially when all patients hope to salvage the involved globes.

Currently, the validated management method of post-traumatic retinal detachment is vitrectomy combined with the implantation of vitreous substitute tamponade [3]. The main tamponades include air, perfluoropropane (C3F8), silicone oil, and perfluorocarbon liquid, with C3F8 and silicone oil being the most commonly used. Patients receiving tamponade made with C3F8 and silicone oil are required to rest in a certain prone position after the operation. C3F8 is an inert gas that is expansible, which means that even a slight excess of gas administered during the procedure may lead to complications such as secondary glaucoma and central retinal artery occlusion. Silicone oil is a kind of polysiloxane with organic side chains [4]. It has adequate viscosity and surface tension and limited expansibility. Although it can effectively seal retinal breaks, it is associated with the risk of serious complications over time (e.g., cataracts, glaucoma, and silicone oil emulsification). Patients are exposed to the risk of developing various complications, including silicone oil-dependent eyes after vitreoretinal surgery when they have ruptured globes with large wounds and loss of ocular contents [5–7]. In the most serious cases, patients may experience eyeball atrophy, which eventually requires eyeball removal or prosthetic eye installation.

Foldable capsular vitreous bodies (FCVBs), as a novel vitreous substitution tamponade, offer a promising new option for ophthalmologists. FCVB is a kind of medical rubber product suitable for long-term tamponade. It provides stable support against the retina, which helps avoid the intraocular toxicity caused by direct contact between the tamponade and the intraocular tissues; it reduces the chances of developing relevant complications caused by the vitreous substitutes while restoring the shape maintenance and support function of the vitreous body of the globe, eliminating the patient’s need for enucleation [5, 8, 9].

However, FCVB has been validated via in vivo experiments [10] and has been widely applied clinically [5, 9, 11–12]. Recently, Hashem Abu Serhan et al. provided the first systematic review of studies reported on FCVB implantation and found that FCVB implantation has been used in the management of various complicated ocular conditions, including severe ocular trauma and silicone oil-dependent eyes. When compared to SO, FCVB showed good visual outcomes, fewer IOP fluctuations, and a good safety profile [13]. In this retrospective study, we evaluated the feasibility of FCVB in the severe case setting via a systematic review of 61 cases of FCVB procedures performed in our hospital during 2018–2019.

## Materials and methods

### FCVB Material

**Fig. 1:**
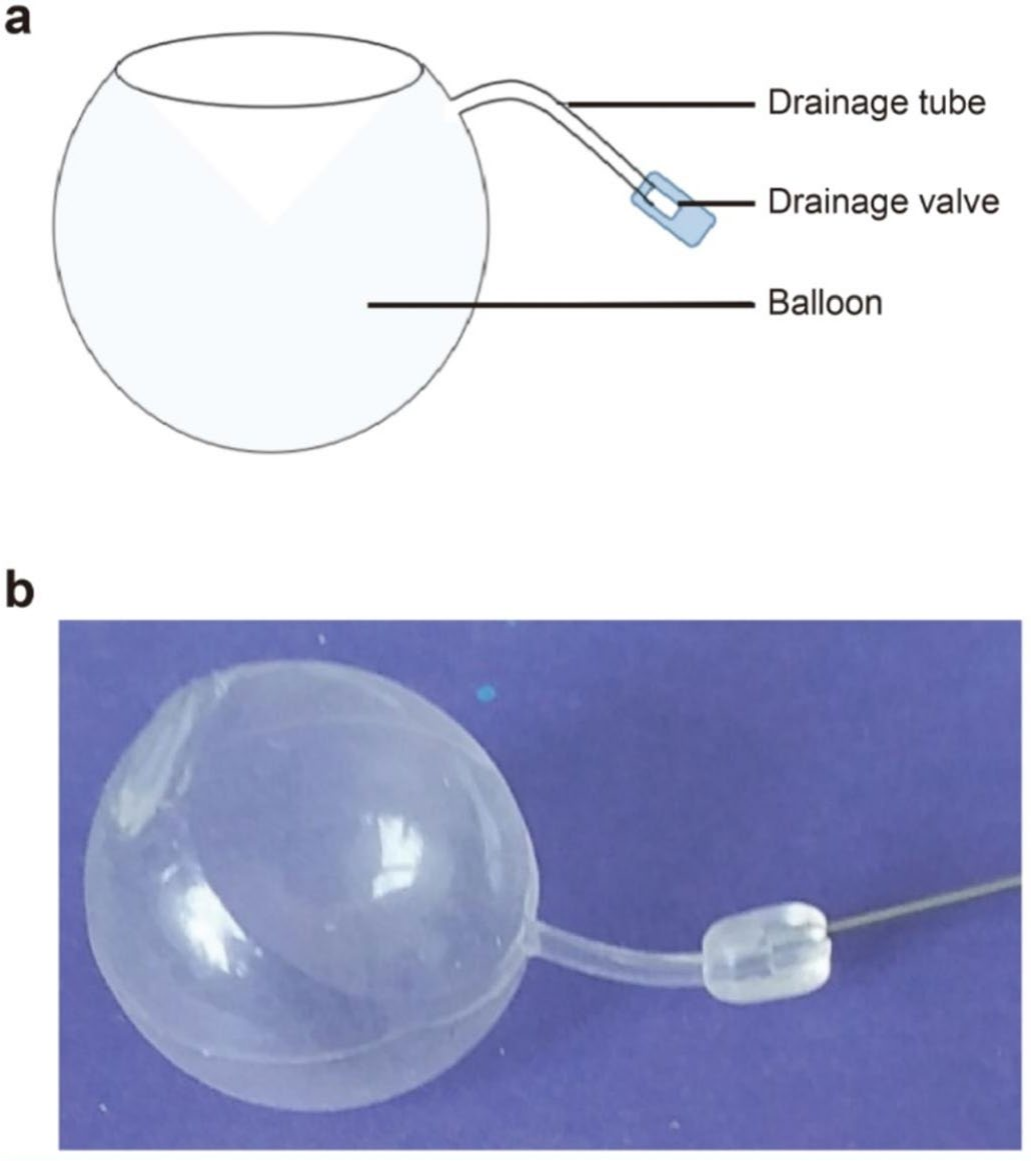
Structural schema of an FCVB. (**a**) An FCVB is comprised of a capsule, a drainage tube, and a drainage valve (**b**) The capsule (inflated)

The FCVB used in these procedures was developed by the State Key Laboratory of Ophthalmology under the Sun Yat-Sen Ophthalmology Center of Sun Yat-Sen University and codeveloped and produced by Guangzhou Vesber Biotechnology. The product received the Chinese medical device registration certificate for clinical use in China on July 27, 2017. This product was designed in the shape of a vitreous cavity and came with a drainage tube and a pressure-adjustable drainage valve, similar to the design of a glaucoma valve (Fig. 1).

### Study subjects

We systematically reviewed 61 patients receiving FCVB implants within one year (5/1/2018-5/31/2019) since FCVB was introduced in the Hebei Eye Hospital; the sample represented 61 eyes (51 males with 51 eyes; 10 females with 10 eyes). The average age of the patients was 44.98 (± 14.60) years old, with the oldest patient being 75 years old and the youngest 8 years old. There were 32 eyes with no light perception, 51 eyes with serious rupture, and 10 eyes with serious, complicated trauma, which became silicone oil-dependent after the operation (Table 1). Patients with severely ruptured globes underwent one-stage suturing and vitrectomy combined with FCVB implantation 9–28 days (mean: 14.22 ± 2.66 days) after the injury. The patients with silicone oil-dependent eyes had their silicone oil tamponade removed to release their intraocular proliferation and deformation before receiving FCVB implantation. There were 43 eyes with ciliary body detachment and 30 eyes with aniridia or iris defects. Before the implantation procedures, the patients and their families were informed about the operation and implants in detail and signed informed consent forms. This clinical trial was conducted following the Declaration of Helsinki and was approved by the Ethics Committee of Hebei Eye Hospital (Ethics Approval Number: HBSYKYYLL2018-02). All patients signed informed consent forms. The inclusion criteria were patients with severe retinal detachment that could not be managed with simple or heavy silicone oil tamponade, including one of the following conditions: (1) severely ruptured globes, with retinal or choroidal defects; (2) large posterior sclera dehiscence, accompanied by choroidal or retinal detachments, which could not be repaired; (3) severe ocular trauma with recurrent retinal detachment after silicone oil tamponade; (4) retinal detachment with stiffening degeneration, which could not be treated with silicone oil alone; (5) axis length of 16–25 mm. Exclusion criteria were defined as being met by (1) patients with known allergy to silica gel or with keloid-prone constitution; (2) patients with severe ocular inflammation; (3) patients with transparent crystalline lens in the eye for operation; (4) patients with a visual acuity of 0.4 or under on the fellow eye; (5) patients having a fellow eye with intraocular surgical history; (6) patients with severe systemic diseases (e.g., diseases involving the cardiovascular, respiratory, digestive, nervous, endocrine, or urogenital systems).

**Table 1:** FCVB size selection, volume of silicone oil and basic information for 61 cases

Case	Age	Gender	Preoperative diagnosis	Size	Volume of Silicone oil (ml)
1	8	M	silicone oil dependent eye	AV-13.5P	3.5
2	33	M	ocular rupture	AV-13.5P	2.2
3	51	M	ocular rupture	AV-13.5P	3
4	75	M	ocular rupture	AV-13.5P	3.7
5	64	M	silicone oil dependent eye	AV-13.5P	3
6	32	F	ocular rupture	AV-13.5P	3.3
7	37	F	ocular rupture	AV-13.5P	2.5
8	53	M	silicone oil dependent eye	AV-13.5P	3
9	48	M	ocular rupture	AV-13.5P	3.5
10	28	F	ocular rupture	AV-13.5P	2.2
11	49	M	ocular rupture	AV-13.5P	3.5
12	34	F	silicone oil dependent eye	AV-13.5P	3.3
13	11	F	ocular rupture	AV-13.5P	3
14	65	F	ocular rupture	AV-13.5P	3.3
15	55	M	ocular rupture	AV-13.5P	4
16	25	M	silicone oil dependent eye	AV-13.5P	3.2
17	56	M	silicone oil dependent eye	AV-13.5P	4
18	47	M	ocular rupture	AV-13.5P	3.2
19	26	M	ocular rupture	AV-13.5P	3.2
20	44	M	ocular rupture	AV-13.5P	3.2
21	50	M	ocular rupture	AV-13.5P	3.3
22	21	M	ocular rupture	AV-13.5P	4
23	56	M	ocular rupture	AV-13.5P	3
24	36	M	ocular rupture	AV-13.5P	3.3
25	17	M	ocular rupture	AV-13.5P	3.4
26	47	M	ocular rupture	AV-13.5P	2.9
27	33	M	ocular rupture	AV-13.5P	3.3
28	60	M	silicone oil dependent eye	AV-13.5P	3.2
29	49	M	ocular rupture	AV-13.5P	3.2
30	66	M	ocular rupture	AV-13.5P	3
31	40	M	silicone oil dependent eye	AV-13.5P	2.9
32	63	M	ocular rupture	AV-13.5P	3.4
33	43	M	ocular rupture	AV-13.5P	3.5
34	54	M	ocular rupture	AV-13.5P	3.5
35	44	M	ocular rupture	AV-13.5P	3.2
36	52	F	ocular rupture	AV-13.5P	3.2
37	52	M	ocular rupture	AV-13.5P	4.2
38	53	M	ocular rupture	AV-13.5P	4.5
39	28	M	ocular rupture	AV-13.5P	3
40	53	M	ocular rupture	AV-13.5P	3
41	49	M	ocular rupture	AV-13.5P	3.2
42	49	M	silicone oil dependent eye	AV-13.5P	2.2
43	50	M	ocular rupture	AV-13.5P	3.2
44	51	M	silicone oil dependent eye	AV-13.5P	3
45	40	M	ocular rupture	AV-13.5P	3.6
46	33	M	ocular rupture	AV-13.5P	3
47	71	M	ocular rupture	AV-13.5P	3.5
48	49	M	ocular rupture	AV-13.5P	3.5
49	50	M	ocular rupture	AV-13.5P	4.2
50	49	F	ocular rupture	AV-13.5P	2.2
51	25	M	ocular rupture	AV-13.5P	3.2
52	53	M	ocular rupture	AV-13.5P	4
53	38	M	ocular rupture	AV-13.5P	2.2
54	48	M	ocular rupture	AV-13.5P	3
55	28	M	ocular rupture	AV-13.5P	3
56	32	M	ocular rupture	AV-13.5P	2.7
57	45	M	ocular rupture	AV-13.5P	3.5
58	33	M	ocular rupture	AV-13.5P	2.7
59	44	F	ocular rupture	AV-13.5P	3.6
60	55	F	ocular rupture	AV-13.5P	2.5
61	51	M	ocular rupture	AV-13.5P	3.7

### Ophthalmology examination

All patients underwent routine preoperative and postoperative examinations in their follow-up clinical visits, which included visual acuity inspection using the international standard visual acuity chart, slit lamp examination, front endoscopic segment inspection, intraocular pressure measurements, AS-OCT, B-scan ocular ultrasound, UBM inspections, corneal endothelial measurements, and AS photography and color fundus photography examinations.

### Operation procedures

**Fig. 2:**
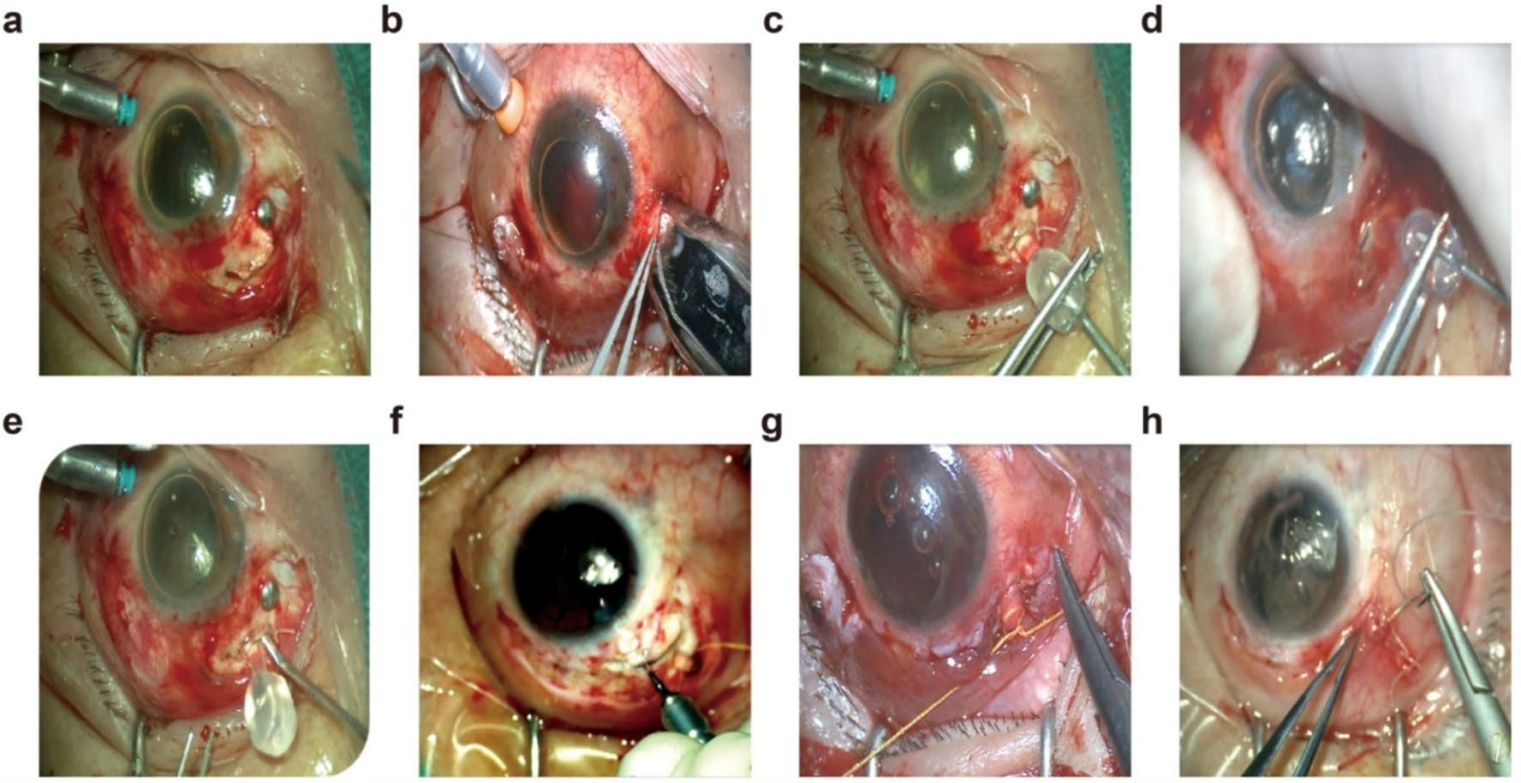
Steps of FCVB implantation. (**a**) Scleral incision construction: 5 mm incision into the limbus followed by a 4-5 mm straight incision, and one 1 mm lateral incision on each side to form an incision of ฺ shape. (**b**) Push the capsule into the eye via a syringe (**c**) Inject silicone oil (**d**) Palpation of the scleral pressure (**e**) Adjust the capsule position (**f**) Flush out the blood in the eye (**g**) Fix the drainage tube (**h**) Suture the subconjunctival tissue and bulbar conjunctiva

All patients underwent vitrectomy. When the vitreomacular traction and proliferative membranes were removed during the operation, the retina’s morphology recovered and was ready for FCVB implantation in the next stage. The capsule was checked for airtightness before it was aspirated to a vacuum state, folded, and loaded into an injector. The intraocular perfusion incision was approximately 3.5 mm from the limbus that was constructed and prolonged, and the capsule was then pushed into the eye in perfusion under the operator’s direct vision. With the lens surface of the capsule positioned upward, the capsule was then fully expanded by injecting silicone oil via the drainage valve until the retina was well supported. The scleral incision was then sutured, and the drainage tube was ligated and sutured to the sclera (Fig. 2).

### Follow-up indicators

The postoperative follow-up ranged from 1 to 36 months, with 61 eyes followed up at 6 months, 61 eyes at 12 months, 57 eyes at 24 months, and 52 eyes at 36 months after surgery. The patients were followed for their visual acuity, intraocular pressure, the state of their cornea and retina, and the position of their FCVB. They were also observed for any ocular inflammation, abnormal intraocular hemorrhage, and/or sympathetic ophthalmia for the safety evaluation of FCVB implants.

### Statistical analysis

Because the poor visual acuity of patients with severe complex ocular trauma cannot be expressed by ordinary visual acuity scale values, they need to be graded and scored according to the best visual acuity: 0 for no light perception, 1 for light perception, 2 for manual, 3 for index, 4 for ≤ 0.1, and 5 for > 0.1. The IOP of some patients cannot be measured and is often measured clinically by the finger-prick method, according to clinical experience IOP ranges: T-2, < 5 mmHg; T–1, 5 ～ 9 mmHg; Tn, 10 ～ 21 mmHg; T + 1, 22～ 34 mmHg; T + 2, 35 ～ 60 mmHg。The IOP was also graded, with a score of 0 for T-2 and < 5 mmHg, 1 for T-1 and 5 ～ 9 mmHg, 2 for Tn and 10 ～ 21 mmHg, 3 for T + 1 and 22～ 34 mmHg, and 4 for T + 2 and 35 ～ 60 mmHg. Some patients were unable to have their corneal endothelial count measured due to corneal rupture, edema, and blood staining. We also developed a grading scale according to the condition of the cornea: 0 for 《800 cell/mm^2^, 1 for > 800-1500cell/mm^2^, 2 for > 1500-2500cell/mm^2^, 3for > 2500-3500cell/mm^2^, and 4 > 3500cell/mm^2^.

In this study, visual acuity, intraocular pressure, and corneal endothelial count were compared between preoperative and final postoperative follow-up scores using the Wilcoxon signed-rank test. Axial length values were expressed as the mean ± standard deviation, and the Wilcoxon signed-rank test was used for the comparison of the affected eye with the healthy eye and for the comparison of the preoperative and postoperative final follow-up scores of the affected eye. The statistical analysis was performed on GraphPad version 9.0 (GraphPad Software, San Diego, CA), and a P < value of 0.05 was considered statistically significant.

## Results

### Operation results

All 61 patients (61 eyes) enrolled in this study received successful FCVB implantation. During the operation, severe retinal detachment with giant retinal tears was observed in all patients. The remaining retina was restored. Thirty-two eyes received retinal photocoagulation. AV-13.5P FCVB was selected for the operation, and the injection volume of silicone oil was 2.2-4.0 mL (mean: 3.22 ± 0.5) (Table 1).

### Changes in visual acuity

Before the operation, 32 affected eyes had no light perception, 19 eyes had light perception, 6 eyes had hand motion vision, and 4 eyes had finger-counting vision. At the last follow-up, 24 eyes had no light perception, 19 eyes had light perception, 9 eyes had hand motion vision, and 6 eyes had finger-counting vision. The difference between the final follow-up visual acuity score and the preoperative visual acuity score was not statistically significant (P = 0.7658).

### Changes in intraocular pressure

Before the operation, 49 eyes had unmeasurable intraocular pressure due to corneal opacity, of which the intraocular pressure of 3 eyes was estimated to be T-2 by finger palpation, 27 eyes were estimated to be T-1, and 19 eyes were estimated to be Tn. The remaining 12 eyes had intraocular pressure in the range of 6–16 mmHg. At the last follow-up, the intraocular pressure of 25 eyes remained unmeasurable because of corneal opacity, among which 7 eyes were estimated to have T-1 level intraocular pressure and 18 eyes were estimated to have Tn. The remaining 27 eyes had IOPs in the range of 10–21 mmHg. The IOP score at the final follow-up was higher than the preoperative IOP score, and the difference was statistically significant (P < 0.0001).

### Corneal changes

**Fig. 3:**
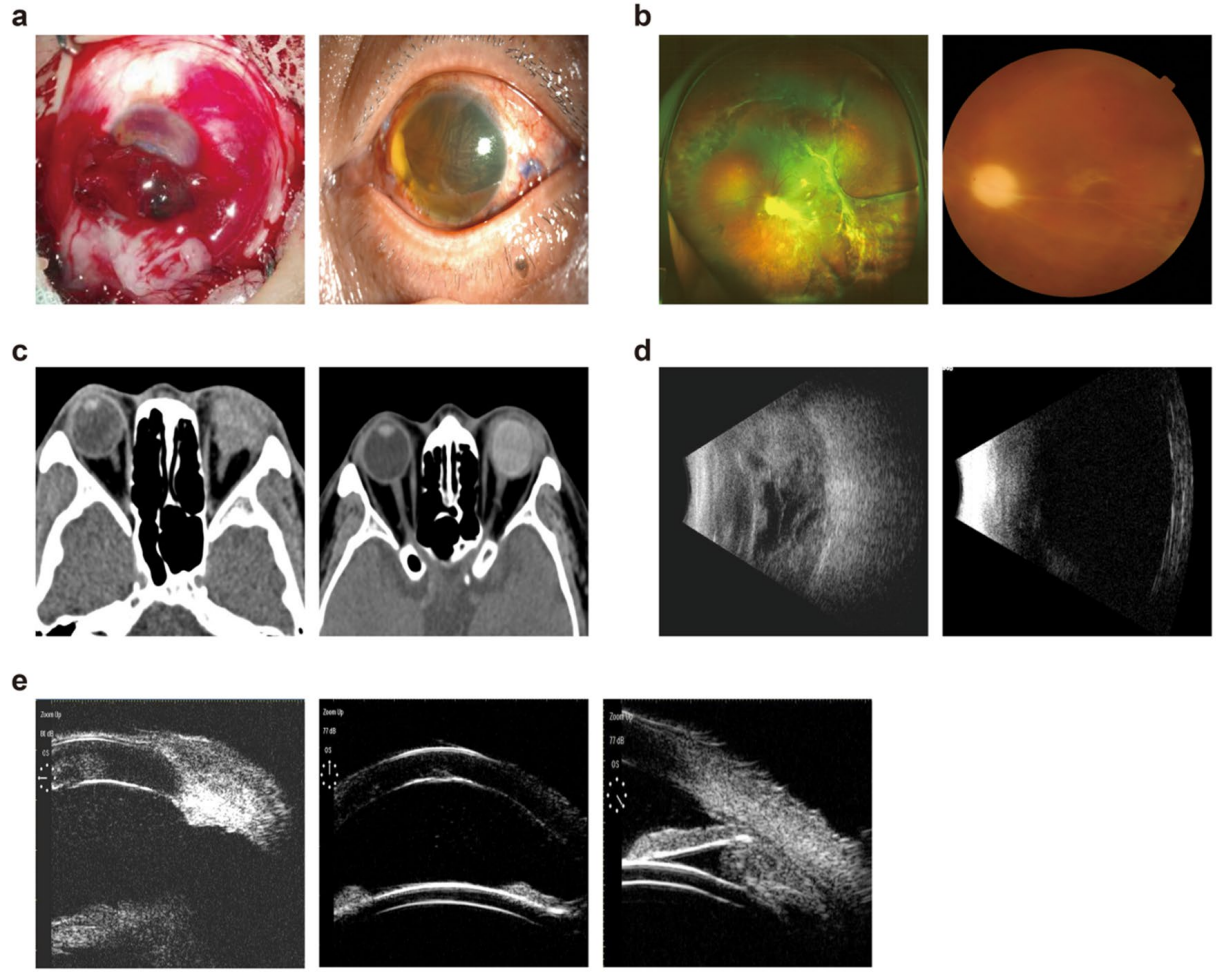
Images of before/after the FCVB implantation. (**a**) Conditions of a cornea with inferior limbal rupture before/after the operation (**b**) Fundus photography after FCVB implantation (**c**) Orbital CT images before and after FCVB implantation (**d**) B-scan images before and after FCVB implantation (**e**) UBM images before and after FCVB implantation

Before the operation, 12 eyes had clear or basically clear corneas, 30 eyes had corneas with localized opacity and measurable corneal endothelial cell counts, and 19 eyes had visible corneal edema or corneal blood stained with unmeasurable corneal endothelial cell counts. The remaining 42 eyes had a corneal endotheliometer range of 775–2845 cell/mm^2^. At the last follow-up, the corneal endothelial cell counts of 22 eyes remained unmeasurable due to corneal opacity. The remaining 30 eyes had a corneal endothelial count range of 557–2078 cells/mm2. The endothelial count score at the final follow-up was lower than the preoperative score, and the difference was statistically significant (P < 0.05). There were no patients with corneal loss at the time of final follow-up, but corneal clouding was worse than before surgery in 32 patients, including 11 with total corneal white clouding, 6 with cosmetic corneal contact lenses, and 5 with thin prosthetic lenses after conjunctival masking (all patients under 45 years of age with high cosmetic requirements) (Fig. 3A).

### Axis changes

Before the operation, the axial length of the affected eye was 22.71 ± 1.69 mm. The axial length of a healthy eye was 23.38 ± 0.9 mm. The mean value of the affected eye was slightly lower than that of the healthy eyes, and the difference was statistically significant (P < 0.001). At the last follow-up, the axial length of the affected eyes was 23.31 ± 0.86 mm, which was improved compared with the preoperative period, and the difference between the two was statistically significant (P < 0.05). There were 11 patients whose axial lengths were at least 2 mm shorter than the reference range before the operation. The difference was as great as 8.68 mm in the worst case. Their intraocular pressures were evidently below the normal range, which indicated globe atrophy. The intraocular pressures of the 11 patients after the operation were higher than before and between 10 and 21 mmHg, and their ocular axes were extended.

### Fundus retinal reattachment

The reattachment status of the patient eyes were assessed according to the results of binocular indirect ophthalmoscopy, B-scan, ocular ultrasound, and color fundus photography. There was complete retinal restoration and no recurrence at the last follow-up (Fig. 3B).

### Status of FCVB after operation

The slit lamp examination showed that the FCVBs were in the correct position. Only 12 patients developed hyperplastic membranes in the anterior capsule. Orbital CT showed complete eye rings and a full globe. B-scans showed that the capsules had homogeneous echoes and a complete shape. UBM revealed normal depths in the patients’ anterior chamber, and the anterior membrane of the capsule had normal reflection, and the FCVBs were in contact but did not compress the ciliary bodies (Fig. 3C-E).

### Patient safety evaluation

No broken capsules or silicone allergies were reported in the patients. Except for a few cases of mild to moderate conjunctival hyperemia and aqueous flares, no obvious ocular inflammation occurred in the patients during the follow-ups. No intraocular hemorrhage, silicone oil emulsification, or sympathetic ophthalmia was found in the patients at the time of final follow-up.

## Discussion

Severe ocular trauma is one of the important causes of uniocular sight loss [14] worldwide. Globally, there are 180,000 patients, with approximately 33,000–50,000 of them children [15]. In the management of ocular trauma, one of the fundamental issues facing ophthalmologists worldwide is to restore structural integrity in time to salvage the damaged globe.

In this study, we explored the application of foldable capsular vitreous bodies in the management of severe ocular trauma. We found no evident changes in the 61 patients reviewed after FCVB implantation, which suggested that even though the FCVB could provide continued support to the retina and hold it to its normal anatomical position, it could not reverse the damage to the ocular tissues, especially damage to the optic nerve and retinal posterior poles, and thus could not restore the visual functions of the eyes involved. This further demonstrated that FCVB serves only to restore the normal shape of the globe without restoring or improving visual acuity and was thus consistent with previous findings [16]. In addition, some patients may experience aggravated corneal opacities after implantation or develop hyperplastic membranes around the capsule, both of which may compromise their vision.

Intraocular pressure is an important factor in maintaining the homeostasis of the eye. Severe ocular trauma may result in damage to the iris and the ciliary body or loss of ocular contents due to a ruptured globe, affecting intraocular pressure [17]. In our research, we found that the intraocular pressure of 91.8% of patients remained in the normal range after the operation. Their UBM images showed that the FCVB did not compress the ciliary body and that the function of the ciliary body remained unaffected. This illustrated that FCVB can maintain the shape of the posterior chamber to allow the aqueous humor circulation to be slowly recovered until the ciliary body function is restored on its own. However, further investigations are needed to determine whether the supporting function of the FCVB would be sufficient to maintain the intraocular pressure of patients with severe ciliary body defects and avoid low intraocular pressure caused by decreased aqueous humor secretion.

Trauma to the eye may lead to injuries to various ocular structures, including the cornea, one of the most frequently damaged sites. Repeated operations after trauma are also accountable for the loss of corneal endothelial cells [14, 18]. We found that the patients’ corneal endothelial cell count had decreased after the operation, but no patients reported corneal endothelial decompensation by their last follow-up clinical visit. During the follow-up window, 52.5% of patients experienced aggravated corneal opacity, with 11 of them reaching the state of corneal leukoma and losing light perception. The cause of corneal opacity may be severe damage to the corneal endothelium caused by large corneal and/or corneal limbal wounds. Localized corneal opacity or severe corneal edema occurred after the first-stage suturing. It was speculated that the implantation of FCVB may have caused detrimental effects on the metabolism of nutrients in the aqueous humor, leading to insufficient corneal nutrient supply and consequentially postoperative corneal opacity and even corneal leukoma. Due to the short follow-up window, further exploration is required to determine whether FCVB may lead to bullous keratopathy.

Severely ruptured globes are often accompanied by grave damage to the ciliary body, retina, and choroid. During the procedures to manage ruptured globes, it is often observed that the affected eyes have giant retinal tears or proliferative contractile cellular membranes that are difficult to flatten, detached choroids that cannot be reset, or persistent low intraocular pressure after the operation. All these factors made it difficult to remove the silicone oil in the eye after the operation, which led to the development of silicone oil-dependent eyes [19]. Long-term exposure to silicone oil may lead to complications, including intraocular toxicity and silicone oil emulsification. Regular replacement of silicone oil is needed, which in turn eventually inflicts band keratopathy and/or global atrophy, resulting in inevitable enucleation [20]. FCVBs have excellent mechanical and optical properties and biocompatibility with human eyes. They are designed to mimic the vitreous cavity. During the implantation procedure, the capsule is injected into the vitreous cavity and inflated by the injection of silicone oil. Afterward, the inflated capsule can effectively support the maintenance of the morphology of the globe and the intraocular pressure. No special postoperative position is needed. Since the silicone oil in the capsule will not be in direct contact with the aqueous humor, the silicone oil is unlikely to be emulsified [5, 21]. Among the patients reviewed in this study, the retinal reattachment rate after FCVB implantation was 100%; this was higher than the observed 73–89.6% reattachment rate of post-traumatic retinal detachment treated with vitrectomy combined with inert gas or silicone oil tamponade in previous studies [22–24].

Ruptured globes are threatening conditions that in the worst cases may lead to structural disorder of ocular tissues, leading to massive leakage of eye contents and eventually globe atrophy and even enucleation [25]. In our research, we identified 11 patients who had severely ruptured globes with giant tears and massive loss of eye contents. Their eyes showed obvious dents before the operation. The B-scan showed clear patterns of globe atrophy. After FCVB implantation, their intraocular pressure returned to the normal range, the axial length of their eyes extended, the eye globe returned to a full shape, and atrophy was controlled, which negated the need for eye removal. The control rate of globe atrophy in this study was 100%, and none of the 61 patients had eyeball enucleation, which was significantly below the post-trauma enucleation rate of 11.8–41.8% in studies outside of China [26]. Although there were 11 patients who underwent conjunctival patching due to corneal leukoma, their eyeballs remained in good shape, and they had undergone the procedure for cosmetic reasons.

When a rupture is not managed with care in time, it can easily cause endophthalmitis in the affected eye and even lead to sympathetic ophthalmia in the healthy eye [25]. In our research, there was no report of rupture of the FCVB allergies to silicone, intraocular infection, intraocular hemorrhage, silicone oil emulsification, or sympathetic ophthalmia. This could fully demonstrate the safety of this technique and its contribution to alleviating the patient’s suffering and improving their postoperative quality of life.

In summary, by reviewing cases of application of FVCB in complex, refractory vitreoretinal diseases such as retinal detachment caused by trauma, we have found evidence to support its safety and efficacy in maintaining the morphology and intraocular pressure of post-traumatic eyeballs while eliminating the need for special postoperative positions and avoiding complications such as secondary glaucoma, band keratopathy, or the displacement of the silicone oil tamponade to other tissues. This procedure can effectively salvage damaged eyes and negate the need for enucleation, which could inflict inevitable psychological and physical damage on the patient. However, we have yet to verify the longest duration that FVCBs can safely stay in the eyes, which will be subject to further investigation.

## Data Availability

The data used to support the findings of this study are available from the corresponding author upon request.
